# A randomized controlled trial comparing lifestyle intervention to letrozole for ovulation in women with polycystic ovary syndrome: a study protocol

**DOI:** 10.1186/s13063-018-3009-5

**Published:** 2018-11-16

**Authors:** Dylan A. Cutler, Alana K. Shaw, Sheila M. Pride, Mohamed A. Bedaiwy, Anthony P. Cheung

**Affiliations:** 1 Division of Reproductive Endocrinology and Infertility, Department of Obstetrics and Gynaecology, University of British Columbia, Vancouver, BC Canada; 2 Grace Fertility and Reproductive Medicine, 210–604 West Broadway, Vancouver, BC V5Z 1G1 Canada

**Keywords:** Polycystic ovary syndrome, Ovulation, Lifestyle, Infertility

## Abstract

**Background:**

Polycystic ovary syndrome (PCOS) affects between 8 and 18% of women and is the leading cause of female anovulatory infertility. Unfortunately, common treatments for women trying to conceive can be ineffective as well as disruptive or harmful to patients’ quality of life. Despite evidence that women with PCOS have expressed the need for alternative fertility treatments, lifestyle interventions incorporating a nutritional plan with supplementation, increased physical activity, and techniques for stress management have not been combined as a program and studied in this population. Literature suggests that each of these individual components can positively influence reproductive hormones and metabolic health.

**Methods/design:**

This is a randomized controlled trial which will include 240 women diagnosed with PCOS, according to the Rotterdam criteria, who are trying to conceive. Participants will be randomized to either a comprehensive lifestyle intervention program or prescribed an oral fertility medication, letrozole. These two groups will be further randomized to consume either myo-inositol or a placebo. Participants will be between the ages of 18 and 37 years. Exclusion criteria include women who have already begun fertility treatment, who are currently using myo-inositol or have taken it within the past 3 months, or who are being treated for, or have a history of, an eating disorder. The primary outcome will be the ovulation rate, the secondary outcome will be conception. Other outcomes include miscarriage rates, validated rating measures of overall quality of life (including social, relational, mind/body and emotional sub-categories) and mental health scores (depression, anxiety, and stress).

**Discussion:**

This trial will determine the effectiveness of a structured lifestyle-based comprehensive intervention program for women with PCOS experiencing infertility. In addition, it will determine whether supplementing with myo-inositol provides any further benefit. The objective of this study is to assess a possible non-pharmacological solution to ovulatory dysfunction in these patients and perhaps improve other associated features of PCOS.

**Trial registration:**

ClinicalTrials.gov, ID: NCT02630485. Registered on 15 December 2015.

**Electronic supplementary material:**

The online version of this article (10.1186/s13063-018-3009-5) contains supplementary material, which is available to authorized users.

## Background

Polycystic ovary syndrome (PCOS) is a heterogenous endocrine syndrome characterized by chronic anovulation and hyperandrogenism (clinical or biochemical) according to the original 1990 NIH criteria. The 2003 Rotterdam criteria added the presence of polycystic ovarian morphology on ultrasound which resulted in four distinct PCOS phenotypes. PCOS is also associated with obesity, insulin resistance, metabolic syndrome, reduced quality of life, increased anxiety and higher rates of depression [1]. Between 70 and 80% of women with PCOS have difficulty conceiving due to oligo- or anovulation. Despite ovulation rates of 75–80% achieved with the use of ovulation-induction agents and a conception rate per cycle of 22% (comparable to that of a healthy women trying to conceive naturally in their first year), some women with PCOS remain unresponsive [2, 3]. In contrast, evidence suggests that lifestyle (diet, activity and stress management) influences ovulation and the effectiveness of fertility treatments [4–6].

While lifestyle intervention is recommended as first-line management for women with PCOS and obesity, 45% of women with PCOS reported that they have never received information on lifestyle management and only 61–76% of reproductive endocrinology and infertility obstetric gynecologists (REI-ObGyn) and 46–61% of obstetric gynecologists (ObGyn) recommended lifestyle modifications to patients for fertility or non-fertility problems [7, 8]. Even with efforts to improve dietary and exercise habits, women with PCOS often have difficulty managing their weight. In addition, 62% of women with PCOS reported never receiving information about emotional support or counseling [7]. It appears that attention to general physical and psychological health is inconsistent at the clinical level yet recognized as an important factor in the effective management of women receiving the life-altering diagnosis of PCOS and/or infertility [9, 10].

A survey of 657 women with PCOS found that 99% of participants would prefer to use alternative methods of treatment than those usually prescribed by their physicians such as clomiphene or oral contraceptives [11]. More recently a literature review by Arentz et al*.*, reported that 70% of women with PCOS use a variety of complementary and alternative medicines, such as vitamin, mineral and herbal supplements. While the most common reason given was to “treat PCOS,” other reasons included “to treat infertility,” “to improve general well-being,” and “to reduce depression” [12].

There have been no studies to date evaluating the reproductive outcomes of a comprehensive, three-component, intervention program incorporating structured nutritional and physical activity combined with techniques for stress management in women with PCOS trying to conceive [13]. The literature shows that women with PCOS can significantly benefit from lifestyle changes, specifically, eating a low-glycemic diet, incorporating nutritional supplements, increasing their activity level, and managing stress [14–17]. However, prior lifestyle intervention studies have limitations including high drop-out rates, lack of defining specific PCOS phenotypes, and small cohort sizes. In addition, most studies have focused on only one lifestyle-related change, such as diet, as opposed to a more synergistic approach wherein diet, exercise, and stress reduction are combined. In addition, the implementation of a mind/body program to help reduce stress which could potentially increase ovulation and conception has yet to be studied in a PCOS cohort. Cognitive behavioural therapy has been implemented in infertile populations for over 30 years [18]. These programs teach a variety of coping skills such as learning relaxation techniques, stress management, and provide group support. The relaxation response is a powerful tool already proven effective in stress-related diseases such as cancer, cardiovascular disease, and mental disorders. Since women with PCOS have a higher prevalence of mental health conditions, inducing the relaxation response through mindfulness training could prove an effective stress management strategy for our study cohort [19].

In addition to a low-glycemic diet, physical activity, and stress reduction, a diet supplemented with myo-inositol may provide further hormonal homeostasis and improve metabolic functioning [20]. Myo-inositol is one of nine stereoisomers of inositol and sometimes considered a member of the B complex vitamin family, albeit humans can synthesize this isomer from glucose. It acts as a second messenger in insulin signaling pathways. Several studies have shown that insulin resistance with its compensatory hyperinsulinemia play a pivotal role in both the metabolic and ovarian hormone dysfunction observed in PCOS women. There is evidence that women with PCOS and insulin resistance could be deficient in myo-inositol which would impact glucose metabolism. In addition, myo-inositol levels in follicular fluid are lower in PCOS women with hyperinsulinemia compared to healthy women [21, 22]. An earlier study described that higher levels of myo-inositol in follicular fluid correlated with oocyte quality and maturity [23]. When supplemented with myo-inositol, women with PCOS have experienced reduced serum insulin, testosterone, and increased rates of ovulation [24, 25]. Myo-inositol has also been shown to improve oocyte number and quality [26].

We hypothesize that implementing a comprehensive program of lifestyle changes in women with PCOS will help restore ovulation by weight loss, reduce serum androgen levels and increase sensitivity to insulin. A lifestyle change program could also ameliorate hirsutism over time. Such changes should improve overall psychological well-being and quality of life. Finally, we expect that distinct phenotypes of PCOS will respond differently to lifestyle intervention. Such information will allow us to better determine which phenotypes are best served using our comprehensive approach, and to identify more specifically those factors affecting outcomes.

The objectives of this study are: to determine whether the comprehensive lifestyle intervention described is effective in restoring ovulation and compare this to letrozole; to determine whether the addition of myo-inositol improves ovulation rates, insulin resistance, metabolic parameters; and finally to evaluate differences in the responses for each of four main PCOS phenotype sub-groups.

## Methods/design

### Overall study design

**Fig. 1:**
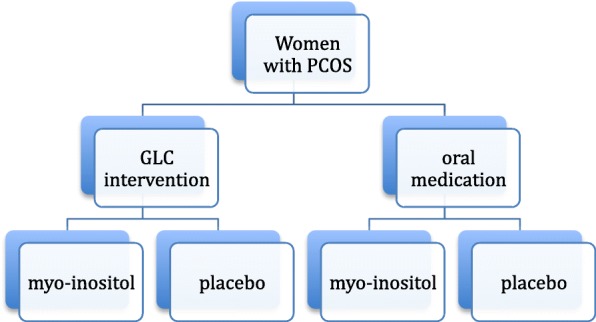
Participants will be randomized into two groups: the “Graceful Lifestyle Changes” (GLC) group and the oral medication group. Both groups will be further randomized to receive either myo-inositol or its placebo

**Fig. 2:**
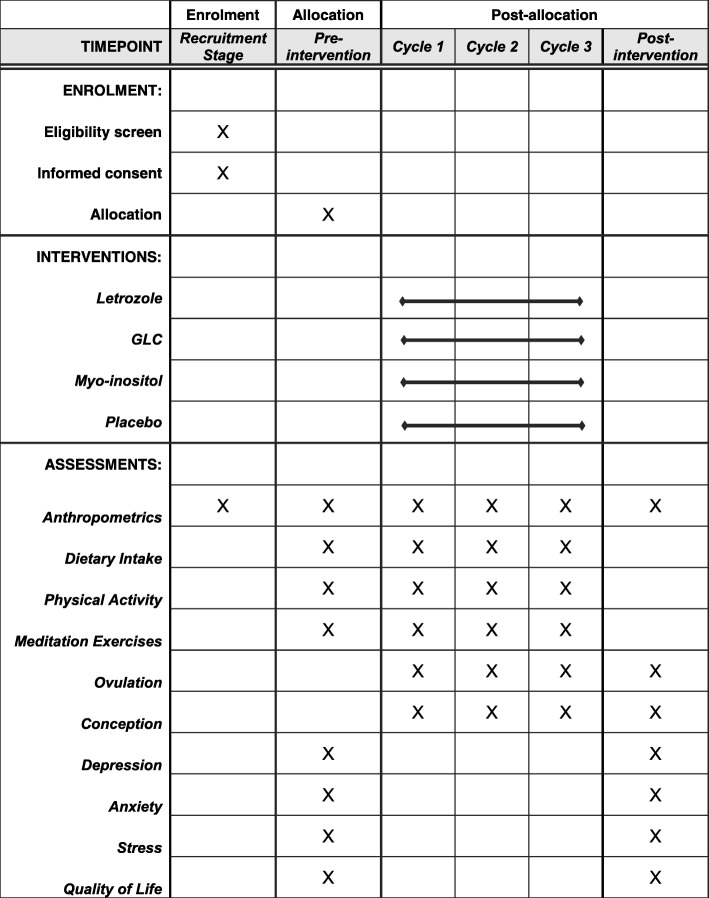
Schedule of enrolment, interventions, and assessments

Women diagnosed with PCOS will be recruited at the Grace Fertility and Reproductive Medicine Centre in Vancouver, Canada. This study follows a factorial design (Fig. 1). Participants will be randomized with a 1:1 allocation into two groups: the first group participating in a comprehensive lifestyle intervention program provided by our clinic (“Graceful Lifestyle Changes” (GLC) program) while the second group will be prescribed letrozole to induce ovulation, with the standard physician counseling (Fig. 2). Within each group, participants will be further randomized with a 1:1 allocation to either receive oral myo-inositol or a placebo. Block randomization will be performed at both stages using a computer-generated random numbers table and the software program REDCap. All clinicians, research scientists and participants involved in the study will be blinded to block sizes and allocation of myo-inositol/placebo. A researcher not involved in the study will be responsible for the randomization procedures. A data monitoring committee will not be necessary given the known safety of the proposed interventions. The SPIRIT 2013 Checklist for trial protocols has been included (see Additional file 1).

### Lifestyle intervention group (GLC)

The GLC group will have weekly check-ins with physicians and educators for 12 consecutive weeks. A wellness booklet designed and provided by clinicians will outline the main concepts being taught. Each week, subjects will receive detailed comprehensive instructions on how to follow a low-glycemic meal plan, incorporate walking 10,000 steps a day, and induce the relaxation response using cognitive behavioural therapy techniques. The low-glycemic-meal plan requires participants to consume 45% of carbohydrates in their diet and no more than 55 g of glycemic load a day, which is similar to previous low-glycemic diet intervention studies. Lists will be provided outlining foods that are low, medium, or high in their glycemic load. Hence, within their overall meal plan, participants will be advised to eat predominantly low-glycemic foods and to limit high-glycemic foods. They will also be provided with a pedometer to record the number of steps taken each day. These steps can be achieved by any form of exercise that participants can sustain. Lastly, participants will practice relaxation response exercises for 20 min daily on their own, in combination with 6 weeks of mindfulness training provided within the 12-week intervention program.

### Data collection

Participants will complete a 3-day food diary report during the baseline, 4th, 8th, and 12th weeks. Participants will also receive a phone call from a trained researcher to complete a 24-h diet recall at the end of the 2nd, 6th, and 10th weeks. This recall will assess all food consumed in the previous 24 h. These combined methods will aim to assess overall compliance to the low-glycemic diet, provide researchers with information on the length of time that participants take to comply, evaluate any fluctuations in eating habits and compare these two types of nutritional assessments.

Participants will also record their physical activity during the baseline, 4th, 8th, and 12th weeks based on their pedometer recordings and will record the meditation and relaxation exercises performed daily.

Compliance to the physical activity portion will be measured through daily pedometer readings recorded by the participant. Compliance will be ensured additionally through weekly check-ins/weigh-ins and frequent motivational email reminders.

Psychological well-being (depression, anxiety, stress, and quality of life) will be assessed before and after the 12-week intervention by two validated self-report questionnaires: the Depression Anxiety Stress Scale (DASS) and the Fertility-Related Quality of Life tool (FertiQoL) [27, 28].

### Letrozole group

The use of clomiphene citrate has been commonly used to treat anovulatory infertility. However, clinical experience and recent study evidence has suggested that letrozole is more effective than clomiphene citrate in achieving ovulation in PCOS [29]. Additionally, letrozole does not negatively affect endometrial thickness. The initial dose will be 5 mg daily for 5 days which can be increased to a total daily dose of 7.5 mg, if necessary, depending on the ovulatory response as per current clinical practice at the Grace Fertility Centre. The first dose will be defined as “cycle day 3” which corresponds to cycle day 3 of spontaneous menses or day 3 of an induced withdrawal bleed following progestogen withdrawal. This treatment regimen will continue for three cycles or until pregnancy is achieved. Subjects will be advised to have intercourse every other day during the expected ovulatory period (beginning cycle day 12, for a week) and to use urinary ovulation prediction kits.

### Myo-inositol group

Participants will be instructed to ingest 6 g of myo-inositol (or its placebo) in water each morning for 12 weeks.

### Outcomes

Ovulation is the primary outcome (categorical “yes” or “no”) and frequency of ovulation (nominal “0”, “1”, “2”, or “3”) during the 12-week (84-day) study. The study length allows observation of three potential ovulatory cycles. The upper limit of a normal ovulatory cycle length is 35 days, hence, to document ovulation using progesterone levels in the expected luteal phase, the study may extend to the end of week 14. Ovulation will be identified by assessing the serum progesterone level on day 22 and, if appropriate, will be repeated 1 week later. A progesterone level greater than 10 nmol/L will be used to confirm ovulation. The secondary outcome is conception (categorical “yes” or “no”). Other outcomes that will be evaluated are miscarriage rates, changes in numerical scores of depression, anxiety, stress, and quality of life based on the DASS and the FertiQoL.

## Statistical methods

The participants’ biochemical and hormonal profiles, physical attributes (body mass index (BMI), hirsutism), ovulatory response, pregnancy status, scores for depression, anxiety, stress and quality of life will be assessed for statistical normality. Each main effect will be compared by analysis of variance (ANOVA) or its non-parametric equivalents. The primary comparison will be between GLC and letrozole. The observed effects of myo-inositol alone are small in clinical experience and there are no reported interactions between myo-inositol and ovulation-induction medications or lifestyle change in the literature. Hence, our power calculations (see below) have been based on the two main effects acting independently. However, our factorial design will provide an opportunity to explore potential interactions (acknowledging the sample size required would change in the presence of interactions) [30, 31]. Categorical data will be compared by chi-square statistics. Differences in dietary intake and dietary composition over time will be compared by ANOVA or its non-parametric equivalent with time as the repetitive measure. Regression analysis will also be performed to determine the individual impacts of diet, activity, and mindfulness on ovulation. In addition, membership in the four PCOS sub-groups will be further explored as a potential predictor for ovulation rates in regression analysis after adjusting for BMI and age.

### Sample size and power calculations

Assuming no interactions between the two main effects as discussed earlier, the target sample size will be 240 participants (120 participants in the GLC group and 120 participants on oral medication). This is based on: a power calculation using a 5% significance level, 80% power comparing two proportions, and current knowledge of the average ovulation rates of lifestyle interventions and letrozole, our primary comparison. We explored a number of combinations of the ovulation rates from letrozole and lifestyle interventions and chose the most conservative estimate to ensure that our sample size was adequate, i.e., 91 in each group. By enrolling 120 participants in each group, we have allowed for a drop-out rate of 32%. Table 1 describes the lowest and highest reported ovulation rates for letrozole and lifestyle interventions, the sample size required in each scenario comparing the two proportions, and the three highest sample sizes required from our calculations, accounting for the length of lifestyle intervention and the start dose of letrozole.

**Table 1:** Sample size calculation based on previous literature

Range of reported ovulation rates	Letrozole ovulation rate	Lifestyle ovulation rate	Total sample size needed
Lowest	62%^a^	38%^b^	130
Highest	86%^c^	67%^d^	150
Median	70%	50%	182

^a^Legro et al. [29]

^b^Palomba et al. [36]

^c^Zeinalzadeh et al. [37]

^d^Karimzadeh and Javedani [38]

When calculating the sample size necessary to assess the effect of myo-inositol on ovulation rates, we reviewed the few published studies available acknowledging that these studies all had small sample size. One randomized controlled trial reported an ovulation rate of 70% when myo-inositol was given to women with PCOS versus a 21% ovulation rate in controls [24]. Similarly, another randomized controlled trial reported an ovulation rate of 65% using myo-inositol in PCOS [32]. Using these rates in a comparison of two proportions, a total sample size of 26 is needed, and easily covered by our original calculation above.

## Discussion

While previous lifestyle-related management programs can improve ovulation rates in some women with PCOS, they have generally focused on weight reduction alone. Legro et al. compared three preconception interventions for women with PCOS prior to undergoing ovulation-induction therapy. The first was lifestyle modification which consisted of caloric restriction, weight loss medication, and exercise. The second was oral contraceptive pills (OCPs) alone, and the third was lifestyle modification in combination with OCPs. The authors found that the two groups incorporating lifestyle modification achieved greater weight loss, higher rates of ovulation, and managed to avoid the onset of metabolic syndrome in comparison to the OCP group [4].

However, restrictive diets resulting in weight cycling is associated with eating disorders (as well as depression) [33]. This is concerning as women with PCOS seem to be at greater risk of developing eating disorders than the general population [34]. Furthermore, long-term weight loss medication is not a sustainable solution for ameliorating symptoms of PCOS.

The intended outcome of this research is the development of a successful comprehensive 12-week program for women with PCOS wishing to conceive. It is evident that women are seeking alternative fertility options that are natural, safe, and effective [35]. While fertility medications can be effective, they are costly and increase the risk of complications such as twin pregnancy. On the other hand, a lifestyle-based program can provide benefits such as weight loss, decreased symptoms of PCOS (decreased hair growth, more regular cycles), improved energy, reduced stress, and better quality of life. Patients frequently drop out of infertility treatment due to factors such as financial/emotional stress and disappointment from failure to conceive despite repeated treatments [18]. Hence, management of PCOS should be more comprehensive and address the multiple factors at play in this condition. This includes not only the endocrine and metabolic perturbations, the anovulatory infertility, but the psychological and emotional impacts of PCOS as well [19].

### Potential biases or limitations

The drop-out rate is often high in lifestyle-based interventions. To mitigate a high drop-out rate we have taken steps such as providing daily email contacts and weekly check-ins between participants and health care providers throughout the duration of the study (12 weeks). A lack of compliance can often become another limitation for intervention studies. To ensure compliance of participants, especially with the lifestyle modification group, we will provide weekly in-person education sessions, as well as periodic check-ins over the phone, in order to explain and reinforce the importance and significance of the recommendations provided and how our study participants might directly benefit from such comprehensive lifestyle changes. These weekly sessions will provide an opportunity for participants to ask questions and receive support from both clinicians and other women struggling with similar issues. After each session, the participant’s weight will be recorded (in a private setting) to help them remain motivated in adhering to the dietary changes and increased physical activity. Several dietary recall methods will be used to measure compliance to the low-glycemic diet, such as a 24-h recall and a food frequency questionnaire, recognizing these tools also have their own limitations.

Although the main goal of this intervention is to achieve a pregnancy, improving overall health and quality of life for women with PCOS is an important component in achieving this primary goal. Infertility per se has a significant psychological impact on women, as does the diagnosis of PCOS. Failed repetitive cycles of hormone therapy further adds to stress and reduced quality of life in PCOS, providing a rationale to develop a comprehensive support program for sustainable lifestyle changes. In addition to improving ovulatory function and the chance of conceiving, such a program has the potential to enhance long-term physical and mental health in this population.

## Trial status

This trial is yet to begin recruitment. Anticipated start date is October 2018.

## Additional file

Additional file 1: Standard Protocol Items: Recommendations for Interventional Trials (SPIRIT) 2013 Checklist: recommended items to address in a clinical trial protocol and related documents. (PDF 119 kb)

## Abbreviations

DASS: Depression Anxiety Stress Scale
FertiQoL: Fertility-Related Quality of Life Tool
OCPs: Oral contraceptive pills
PCOS: Polycystic ovary syndrome
